# Supplementary material for: Transcriptome Analysis and Intraspecific Variation in Spanish Fir (Abies pinsapo Boiss.)
Source: Int J Mol Sci. 2022 Aug 19;23(16):9351. doi: 10.3390/ijms23169351 (PMC9409315; doi:10.3390/ijms23169351)
Supplement: Supplementary file 1 [file ijms-23-09351-s001.zip › Supplementary material.pdf]

Supplementary material

Table S1. Primer list.

Primer Name	Identifier	Gene description	Sequence (5’ – 3’)
qAp_REF1F	*Ap_2641*	Saposin-like aspartyl protease	TTGGATTGCGTATCTTGCTTGC
qAp_REF1R	*Ap_2641*	Saposin-like aspartyl protease	AACATACATFFTTATTGCTTGCCA
qAp_REF8F	*Ap_10901*	Peptidase S24/S26A/S26B/S26C family protein	TGCTCCATTCAGTTGCACCT
qAp_REF8R	*Ap_10901*	Peptidase S24/S26A/S26B/S26C family protein	TGAAATTCCTTGCAGCAGAACA
qAp_1400F	*Ap_1400*	Putative RNA recognition motif domain-containing protein	ACCACCTCGTTCATCATCAGC
qAp_1400R	*Ap_1400*	Putative RNA recognition motif domain-containing protein	GGGGTCTGTTAGCGAAGAGTC
qAp_5633F	*Ap_5633*	Chromomethylase (CMT)	TTCATTCTCGCTCTGGATCCA
qAp_5633R	*Ap_5633*	Chromomethylase (CMT)	GCTAGTCTACTTGGCAGCCTT
qap_14760F	*Ap_14760*	Asparagine synthetase	GTGCAAAGTTGGCGTTTCGTA
qap_14760R	*Ap_14760*	Asparagine synthetase	GCCCATCAAAATACAGCCGTC
qap_22702F	*Ap_22702*	SANT domain-containing protein	TGAAGCTCCTATGTGATGCCA
qap_22702R	*Ap_22702*	SANT domain-containing protein	TGCTGCCAATTCCACATATGC
qap_72505F	*Ap_72505*	Ribulose bisphosphate carboxylase small subunit	CTGAGACACTCTGTTGGGGTC
qap_72505R	*Ap_72505*	Ribulose bisphosphate carboxylase small subunit	AGACGGGCAAAATTGAGCATC
qap_72669F	*Ap_72669*	Red chlorophyll catabolite reductase	TCTGCTCTACTTGGGAATCGTC
qap_72669R	*Ap_72669*	Red chlorophyll catabolite reductase	CTCACAGCCATTTCAAATTGCA
qap_84836F	*Ap_84836*	Cytochrome P450 CYP720B12v2	TCTCTCACGTTTAACCCTGGC
qap_84836R	*Ap_84836*	Cytochrome P450 CYP720B12v2	ATGGCGATCTGAAGTTTTGCG

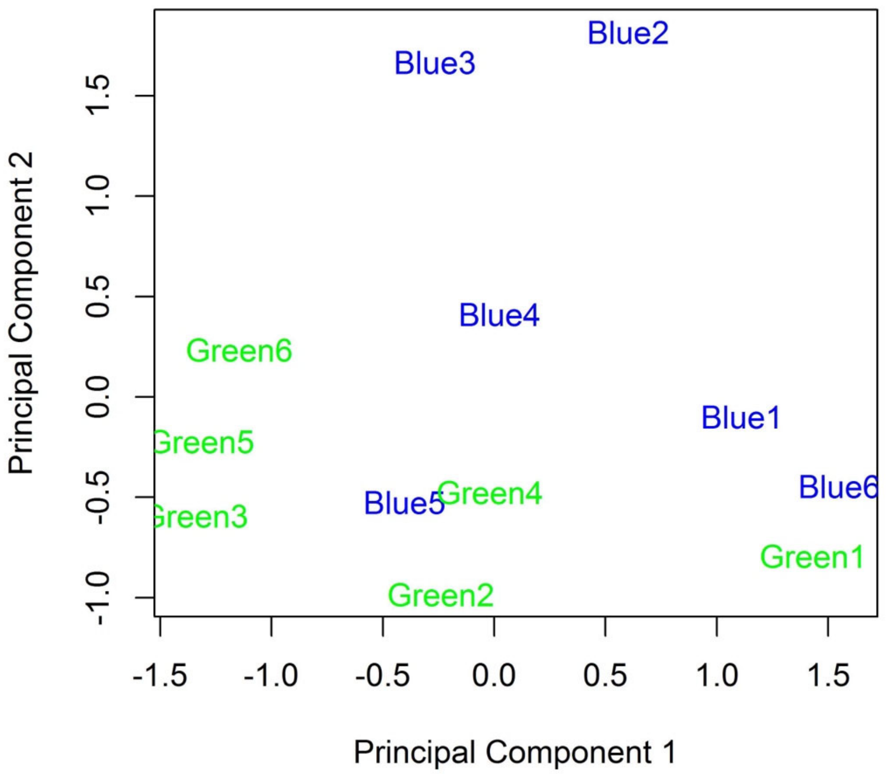

Figure S1. Multidimensional scaling plot (MDS) of needles samples from bluish (B) and green (G) trees.

Dataset S1. RNA-seq differential expression results

Dataset S2. RNA-seq functional enrichment of differentially expressed (DE) transcripts
